# Diagnostic Imaging of Agminated Blue Lesions and Blue Lesions with Satellitosis: Case Series with a Concise Review of the Current Literature

**DOI:** 10.3390/jcm13030894

**Published:** 2024-02-03

**Authors:** Carmen Cantisani, Giovanni Paolino, Antonio Di Guardo, Vito Gomes, Andrea Carugno, Maria Elisabetta Greco, Noah Musolff, Giulia Azzella, Giovanni Rossi, Giuseppe Soda, Caterina Longo, Giovanni Pellacani

**Affiliations:** 1 Department of Dermatology, Sapienza University of Rome, 00185 Rome, Italy; 2 Unit of Dermatology, IRCCS Ospedale San Raffaele, 20132 Milan, Italy; 3 Department of Anatomy and Pathology, Ospedale San Filippo Neri, 00135 Rome, Italy; 4 Dermatology Unit, ASST Papa Giovanni XXIII, 24127 Bergamo, Italy; 5 Ph.D. Program in Molecular and Translational Medicine (DIMET), University of Milan-Bicocca, 20126 Milan, Italy; 6 Department of Molecular Medicine, Policlinico Umberto I, Sapienza University of Rome, 00185 Rome, Italy; 7 Dermatology Department, University of Modena and Reggio Emilia, 41121 Modena, Italy; 8 Azienda Unità Sanitaria Locale–IRCCS di Reggio Emilia, Skin Cancer Center, 42122 Reggio Emilia, Italy

**Keywords:** agminated, blue nevus, melanoma, dermoscopy, metastases

## Abstract

**Background:** Agmination and/or satellitosis in pigmented blue lesions is a phenomenon rarely mentioned in the literature and not well known. This phenomenon can be expressed by several benign and malignant pigmented blue lesions, such as blue nevi, Spitz nevi, melanocytoma and melanoma. On this spectrum, dermoscopy, reflectance confocal microscopy (RCM) and dynamic Optical coherence tomography (D-OCT) represent non-invasive imaging technologies, which may help clinicians in the diagnosis of melanoma and non-melanoma skin cancers in daily clinical practice. **Methods:** Currently, in the literature there is a lack of new data about agminated blue lesions and blues lesions with satellitosis, as well as the lack of a recent and updated review of the literature about this topic. Therefore, considering that clinicians must be confident with the diagnosis of these rare skin lesions, we decided to carry out this work. **Results:** In this paper, four new cases of agminated pigmented cutaneous lesions were described. Moreover, a review of the current literature on this topic was performed. **Conclusions:** A clinical–pathological correlation is often needed to reach a correct diagnosis; currently, dermoscopy and non-invasive diagnostic techniques, such as reflectance confocal microscopy and optical coherence tomography, due to the depth of these skin lesions in the dermis, can only make a partial and limited contribution.

## 1. Introduction

Blue homogeneous pigmentation is a relatively common dermoscopy pattern, found during daily clinical practice. Usually, this dermoscopic pattern does not pose major concerns to expert clinicians, as it is often associated with benign solitary lesions such as blue nevi. However, when pigmented lesions with a homogeneous blue pattern arise as new lesions, they can cause important differential diagnoses with melanoma metastases or primary nodular melanomas.

In addition, some pigmented cutaneous lesions may show multiple lesions arranged in a grouped, linear or specific anatomic area, causing important differential diagnoses. Indeed, while melanoma with loco-regional satellitosis (also in agminated features) is a well-known occurrence, agmination and/or satellitosis in benign pigmented blue lesions is a rare phenomenon, with few cases reported in the literature. Indeed, this aspect can be assumed by several benign and malignant pigmented blue lesions, such as blue nevi, Spitz nevi, melanocytoma and, obviously, in malignant counterparts such as melanoma. On this spectrum, reflectance confocal microscopy (RCM) and dynamic Optical coherence tomography (D-OCT) represent non-invasive imaging technologies, which may help clinicians in the diagnosis of melanoma and non-melanoma skin cancers in daily clinical practice. Specifically, RCM affords a horizontal view of the skin up to the superficial dermis, with a resolution that is almost comparable to conventional histology, while D-OCT allows the evaluation of the skin at high resolution and with no discernible effect on the tissue, providing also an in vivo evaluation of blood vessels and their distribution, with functional information and consequently greater density of data.

For all these reasons, recognizing agminated blue lesions and blue lesions with satellitosis is important for clinicians in order to avoid unnecessary excisions. In this article, our aim was to evaluate the main diagnostic features of agminated benign and malignant lesions to improve their recognition by clinicians. In addition, we performed a concise review of the current literature from 1947 to the current date.

## 2. Materials and Methods

We performed a collection of case series of patients with agminated pigmented cutaneous lesions and/or pigmented cutaneous lesions with satellitosis at the Non-Invasive Diagnostic Imaging and NMSC Outpatient Clinic of the Sapienza University of Rome. For each patient we collected the following data: gender, age, personal medical history, dermoscopic aspects of the pigmented cutaneous lesions, histological features and other non-invasive diagnostic procedures (Vivosight Michelson Diagnostic Ltd. (MDL), Maidstone, Kent, United Kingdom). When available, we also performed a description of histological specimens. Subsequently, we performed a conceptual review of cases of agminated pigmented cutaneous lesions and pigmented cutaneous lesions with satellitosis reported in the literature so far.

A systematic search of the literature was conducted from inception to the 4th of November 2023 in PubMed, EMBASE and Cochrane CENTRAL databases. The following key words were searched on PubMed: (“agminated pigmented cutaneous lesions” [MeSH Terms] OR (“agminated” [All Fields] AND “blue nevus” [All Fields]) OR “disseminated Spitz nevi” OR “Eruptive Spitz nevi” OR “melanoma” [All Fields] OR “melanocytoma” [MeSH] OR “melanoma metastases” OR “cutaneous satellitosis” [All fields] OR “cutaneous metastases” [All Fields] OR “dermoscopy” [All Fields]. For Embase and Cochrane CENTRAL, the following terms were searched: (agminated pigmented cutaneous lesions), (blue lesions satellitosis), (Spitz nevus satellitosis), (melanocytoma satellitosis), (melanoma satellitosis) and (agminated nev*) or melanoma cutaneous metastases or melanocytoma).

## 3. Results

A total of 4 patients (collected at Sapienza University of Rome) presenting with agminated cutaneous lesions or cutaneous lesions with satellitosis were collected. They were 2 female and 2 males, with a median age of 64.5 years, ranging between 38 and 86 years. All patients showed agminated cutaneous lesions or cutaneous lesions with satellitosis showing a blue pattern in dermoscopy, with presence of a whitish veil. Among these lesions there was one case of an agminated blue nevus, one case of a desmoplastic blue nevus and two cases of metastatic melanoma with satellitosis.

**Figure 1:**
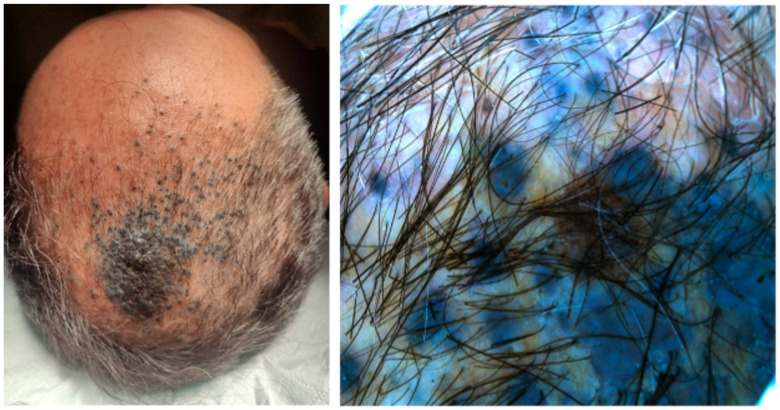
A 5 × 3.5 cm, blue–black plaque on the vertex with multiple satellitosis (**left**) and its aspect on dermoscopy showing homogenous blue–gray pigmentation with yellow hue and satellitosis (**right**).

Case 1. A 53-year-old male patient presented with a recent onset melanocytic lesion in his scalp. His personal and family history for cutaneous malignances were negative. The patient denies systemic symptoms. He was otherwise healthy; he didn’t have any chronic conditions and he was not taking any medication. Clinical examination revealed the presence on the scalp of a blue–black lesion, with plaque-like appearance and measuring about 5 × 3.5 cm (Figure 1). Several few millimeters satellitoses were observed in the right fronto-temporal area. The surrounding skin showed no signs of photoinduced damage. Dermoscopic examination showed homogenous blue–gray to blue–black patterns (Figure 1). Satellitosis were sharply demarcated with a blue structureless pattern.

**Figure 2:**
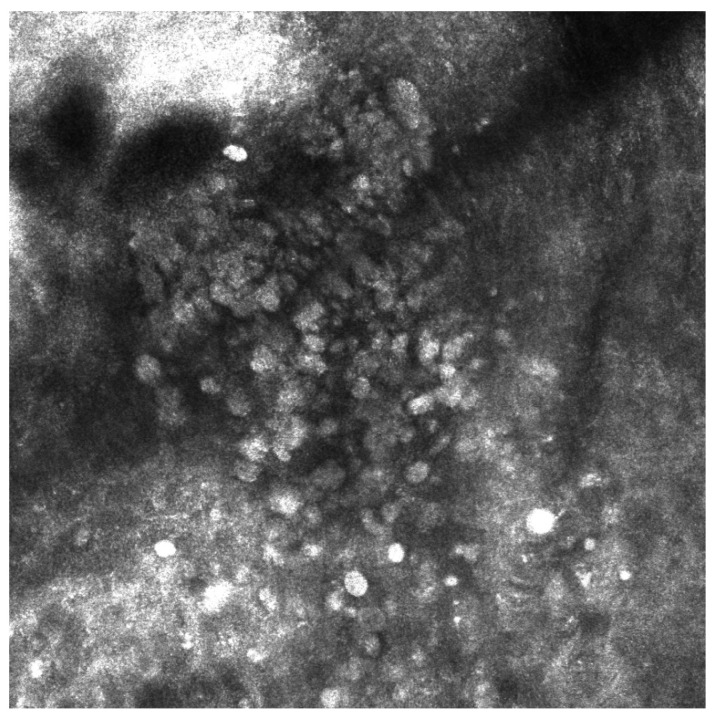
Large hyperreflective pagetoid cells on RCM examination.

**Figure 3:**
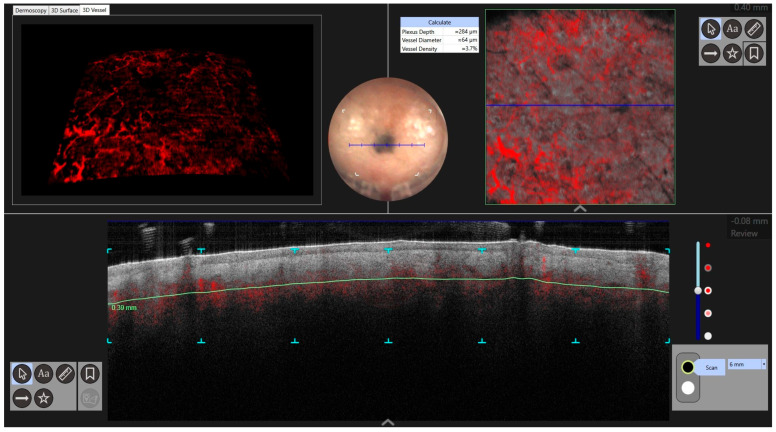
Atypical nests in dermis were found in the DOCT examination of a larger plaque.

**Figure 4:**
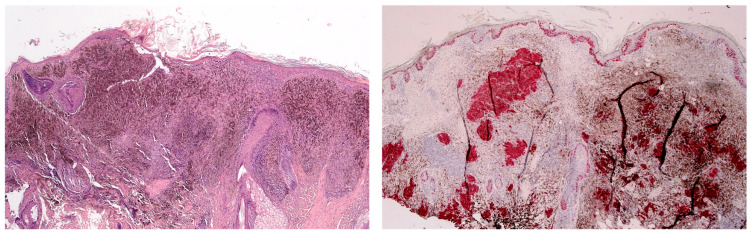
(**Left**) Sections show a dermal tumor that is heavily pigmented. Cellular spindle cell populations are interspersed among melanophages. (Hematoxylin and Eosin, ×40). (**Right**) Immunostaining shows only a part of the cell population is characterized by melanocytes, while the remaining part is made up of histiocytes that have phagocytosed pigment. The immunostaining also shows a proliferation of melanocytes in the basal layer. (Melan A, Mart-1, ×40).

Reflectance confocal microscopic (RCM) examination was performed. It revealed a cerebriform pattern and the presence of large hyperreflective pagetoid cells of a suspected inflammatory nature (Figure 2). Examination of the large plaque on Dynamic-optical coherence tomography (D-OCT) highlighted atypical clusters in the dermis (Figure 3). An incisional biopsy was performed. Histology revealed a dermal component of atypical epithelioid and spindle-shaped cells quantitatively referable to about 30% of the total cellularity (Figure 4). The remaining cell population consisted of melanophageal histiocytes. Melan-A testing showed dendritic elements along the dermo-epidermal junction and along the adnexa. Marked superficial and deep lymphocytic-type inflammation was also present. The Ki-67 test revealed a low expression of melanocytic proliferation. BRAF V600 mutation was detected. A second opinion by another expert histopathologist stated that the diagnosis of melanoma could not be ruled out. The patient was examined for systemic disease through a total-body computed tomography (CT) scan with contrast which was interpreted as negative. At follow-up the lesion went into partial clinical remission. He was treated according to guidelines as locally advanced melanoma.

**Figure 5:**
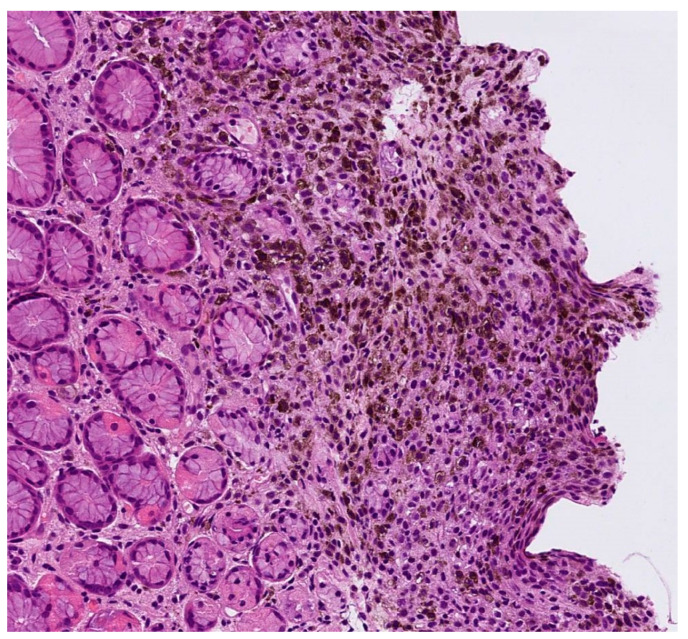
Gastric metastasis from melanoma. Neoplastic cells with intracytoplasmic melanic pigment infiltrate the muscolaris mucosae (hematoxylin and eosin, ×20).

**Figure 6:**
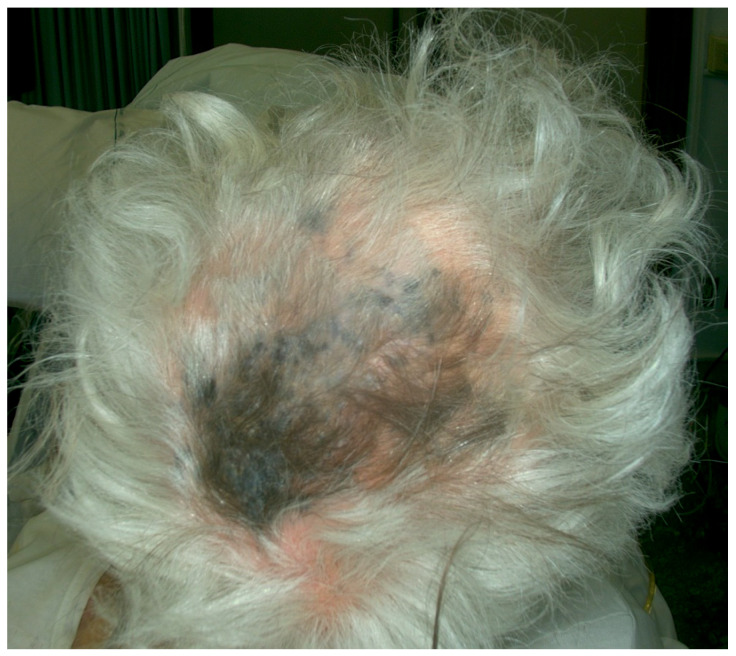
Large blue–black plaque on vertex area, measuring about 6 × 4.5 cm, with multiple satellitoses interspersed between uninvolved skin.

**Figure 7:**
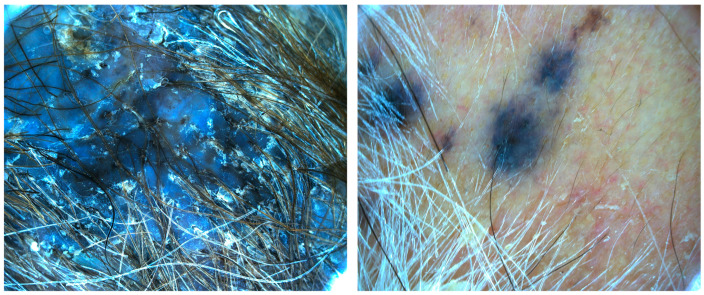
Dermoscopy shows a blue–gray pattern with multiple macular melanocytic lesions and a homogeneous blue pattern.

**Figure 8:**
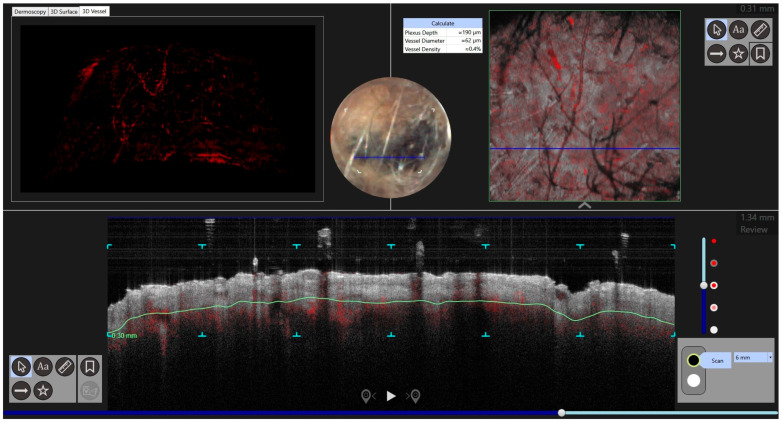
D-OCT in face view shows disarray in the dermal-epidermal junction.

Case 2. An 86-year-old male patient presented for evaluation of hidden melanoma after the finding of peritoneal carcinosis and gastric metastasis from melanoma. The patient had undergone endoscopic gastric biopsy after an episode of gastric bleeding. Histologic examination revealed, at the level of the muscolaris mucosae, a focus of neoplastic cells with a hyperchromic, sometimes vesicular nucleolus and evident nucleolus, nuclear pseudoinclusions and vacuolization of the cytoplasm. Diffuse accumulation of intracytoplasmic melanic pigment was found. Immunohistochemical staining for S100 was positive in neoplastic cells. Histological findings were compatible with metastasis from melanoma (Figure 5). During our dermatologic evaluation the presence of a blue–black plaque of the vertex with several satellitoses all over the scalp was observed. Skin examination showed the presence of a blue–black plaque on his scalp measuring about 6 × 4.5 cm (Figure 6). Dermoscopic examination showed a homogeneous bluish-black pattern in both the main lesion and satellitoses (Figure 7). Optical coherence tomography (D-OCT) examination was performed. D-OCT on the vertical plane showed atypical dermal nests and disarray in the dermo-epidermal junction (Figure 8). The patient died due to the metastatic disease.

**Figure 9:**
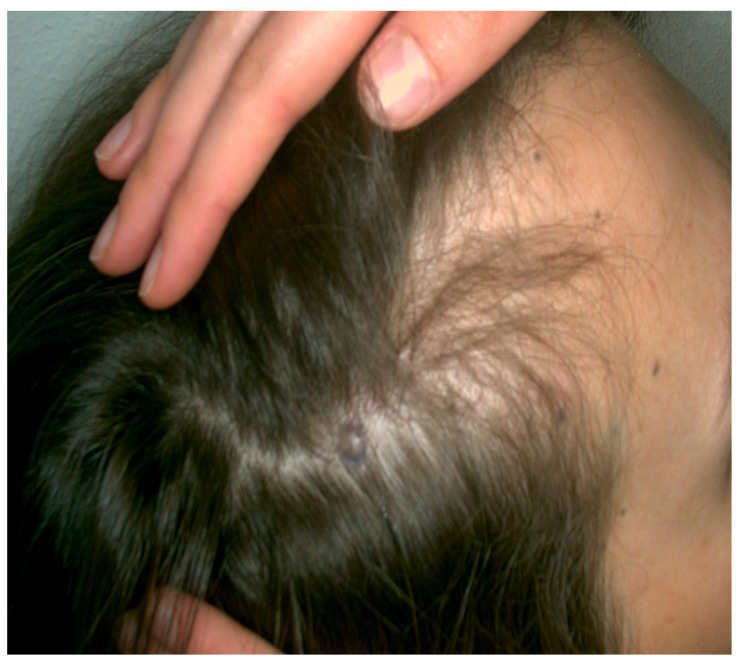
A blue dome-shaped nodule on frontoparietal scalp.

**Figure 10:**
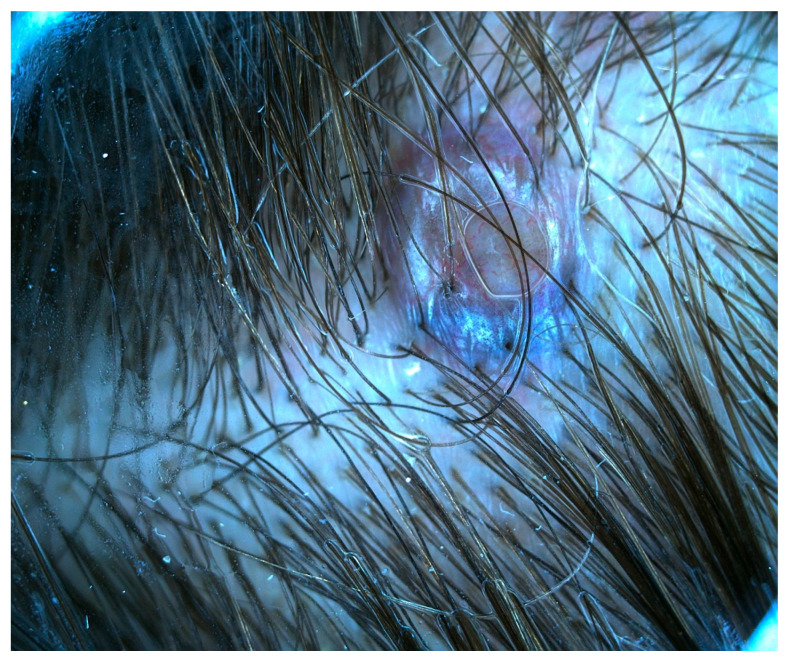
By dermoscopy, violet-blue nodule with thin serpentine vessels.

Case 3. A 38-year-old female patient presented with an asymptomatic blue lesion on her scalp. The lesion increased in size and satellitoses appeared in the last few years after pregnancy. She denied family or personal history of skin neoplasms. Clinical examination showed, in the right frontoparietal area, a blue–black dome-shaped lesion (about 0.6 cm in diameter) (Figure 9). In perilesional skin, a group of 3 blue–black macules, all below 1 cm, was found. On dermoscopic examination, the largest lesion showed a violet–blue color with serpentine vessels (Figure 10), while the satellitoses had a homogeneous blue–brown pattern. Excisional biopsy of the lesions was performed. Histological examination revealed histological features of a desmoplastic blue nevus; at follow-up an absence of recurrence.

**Figure 11:**
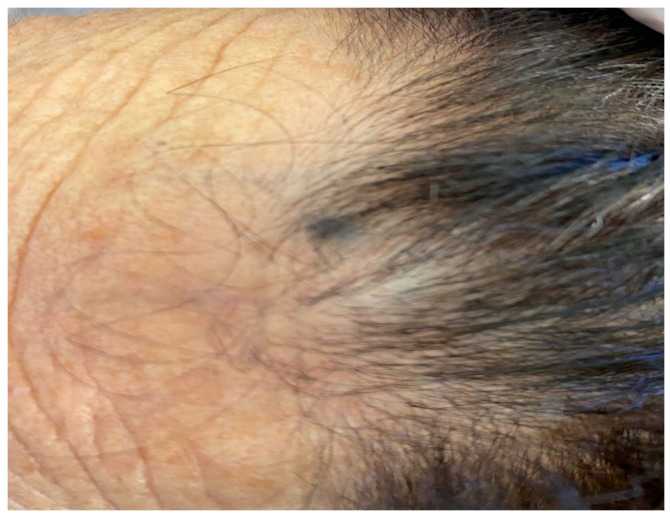
Macular 0.3 × 0.3 cm blue–black lesion on forehead.

**Figure 12:**
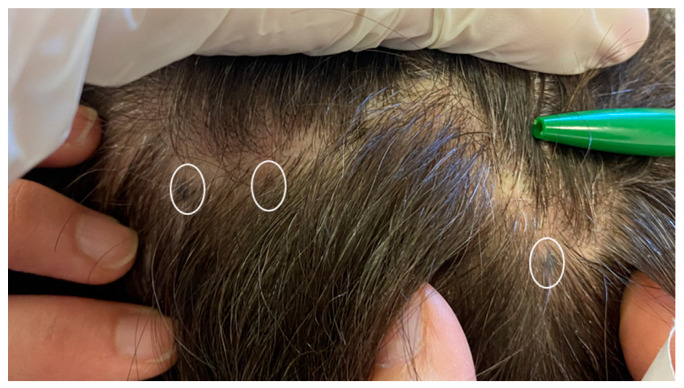
Three brown-black macules interspersed on her scalp (white circles).

**Figure 13:**
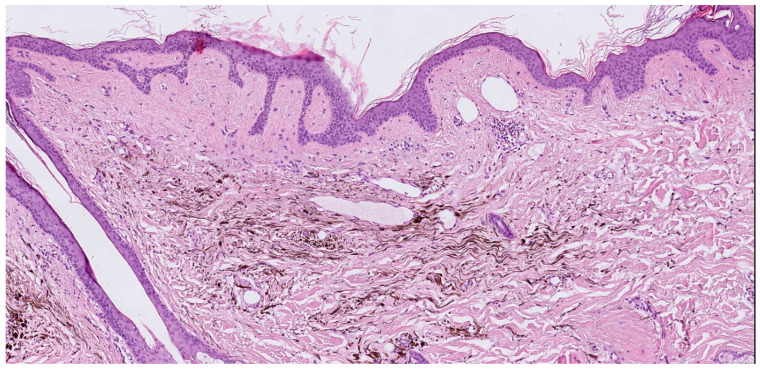
Dermal proliferation of pigmented dendritic spindle melanocytes and collagenous stroma. (hematoxylin and eosin, ×20).

Case 4. A 77-year-old female patient presented with a flat pigmented lesion of the head of recent onset. Her family and personal history was negative for skin malignancies. On clinical examination, a bluish pigmented lesion was found at the frontal site at the hairline, approximately 0.3 cm in diameter (Figure 11). A group of 3–4 blue–brown macules were also found in the vertex area of the scalp, all below 1 cm in diameter (Figure 12). Dermoscopic examination showed a homogeneous pattern of uniform blue–brown color, both for frontal macule and vertex lesions. The frontal lesion was subjected to excisional biopsy. Histological examination showed the histological features of a common blue nevus (Figure 13), which was completely excised. No recurrence was found at the site of surgery. The other lesions did not show any changes in size, color and texture. The clinical and dermoscopic features of all the studied patients are summarized in Table 1.

Finally, as reported above we performed a concise review of the current literature, since 1947 to date, providing clinical and dermoscopic information about agminated blue lesions and blue lesions with satellitosis. All the relevant information is summarized and reported in Table 2.

## 4. Discussion

During daily clinical practice, clinicians may observe blue lesions with atypical features, characterized by single or multiple lesions and/or satellite lesions, posing important differential diagnoses with primary or metastatic malignant blue skin lesions. On this spectrum, agminated blue lesions, agminated Spitz nevus, Spitz nevus with satellitosis and melanocytoma often require important clinical–pathological knowledge by clinicians to be correctly diagnosed, and require performing a correct and accurate differential diagnosis with blue malignant lesions such as metastatic melanoma with satellitosis; we therefore decided to also include this in our series of patients collected for this study.

As reported above, blue lesions usually present as solitary lesions; however, multiple pigmented lesions may appear, causing concerns in clinicians and patients. Agminated lesions appear in a cutaneous area of less than 100 mm and can be congenital or acquired. The term “agminate” derives from the latin word “agminis”, which means “troop”. Since 1947, when the first case of an agminated nevus was reported by Upshaw et al. [1], the clinical aspects of these lesions have been always associated with blue (agminated) nevus; however, this manifestation can also be present in malignant and borderline pigmented lesions, with important differential diagnoses. For this reason, we decided to investigate the clinical, dermoscopic, in some cases confocal microscopy, optical coherence tomography and histologic features of agminated benign and malignant cutaneous lesions in order to focus on some aspects that may help clinicians through a correct diagnosis.

Currently, there are 32 cases regarding agminated blue nevi (ABN) published so far in the literature [2,3,4,5,6,7,10,11,14,15,20,21,22,23,24,25,26,27,28,29,57,58,59,60] (Table 2). The term agminated, by some authors, has also been replaced by other terms such as “plaque-type” or “patch-type” in order to define blue nevi clustered in an area ≤ 10 cm. There is no gender prevalence, with an equal incidence in male and female patients, involving the head/neck region, trunk and extremities. ABN association with GNAQ and CYSLTR2 mutations has been reported in the literature, though in sporadic cases [60]. ABN usually arises as single and solitary cutaneous lesions; however, associations with nevus spilus, exophthalmus, pre-tibial myxoedema, osteoarthropathia (EMO syndrome) and dermatomyositis as well as Darier’s disease have been reported in the literature [2,3,4,5,6,7,10,11,14,15,20,21,22,23,24,25,26,27,28,29,57,58,59,60]. A malignant transformation with a melanoma arising in a previous ABN has been reported [61,62]. Accordingly, dermoscopy can be a useful tool (together with clinical anamnesis of the patients) to reach a diagnosis. Indeed, melanoma metastases may mimic benign blue nevi and vice versa. In dermoscopy, blue melanoma metastases usually show a more homogenous blue pattern, while blue nevi may present multi-component dermoscopic patterns. Indeed, in ABN with multiple lesions (as reported also in our Case 4), a structureless blue pattern is usually associated with brown or grey structureless areas, as well as white/flesh-colored areas [61]. In any case, histological examination confirms the diagnosis, characterized by absence of cellular atypia and other melanoma features in ABN.

Despite Spitz nevi usually present as solitary cutaneous lesions, multiple Spitz nevi can be divided into agminated (ASN) or disseminated [45]. To date, 20 cases of disseminated (or multiple or eruptive) Spitz nevi [45,63,64,65,66,67,68,69,70,71,72,73,74,75,76,77,78,79,80,81,82] have been reported in the literature, while, regarding agminated Spitz nevi, a total of 39 cases have been reported [30,31,32,33,34,35,36,37,38,39,40,41,42,43,44,46,47,48,49,50,51,52,53,54,55,56,83,84,85,86,87,88,89,90,91,92,93,94,95]. While disseminated Spitz nevi are characterized by the presence of multiple and eruptive cutaneous lesions in different anatomic areas (e.g., involving the limbs and trunk), agminated Spitz nevi are characterized by the development of multiple lesions in a localized cutaneous distribution, as reported in our patient. Malignant transformation in ASN have not been reported in the literature so far. The presence of ASN is usually associated with a fusion involving the proto-oncogene receptor tyrosine kinase ROS1 [53]. A complex translocation involving TRPM1, PUM1 and LCK has also been implicated [53]. Usually, ASN arises in children (above all under 5 years); however, it may arise also in adults, albeit rarely [45]. The clinical–pathological correlation plays a pivotal role in reaching a diagnosis, since histological lesions did not show features of atypia. The management of ASN is characterized by surgical excision, despite crizotinib (an ALK and ROS1 inhibitor) being successfully used in ANS [53].

Melanocytoma is a cutaneous tumor that is included in the spectrum of atypical melanocytic neoplasms (AMN), which include: cutaneous melanocytomas, melanocytic dysplasia’s, minimal deviation melanomas, borderline melanomas, melanocytic tumors of uncertain malignant potential (MELTUMP) and spitzoid melanocytic tumors of uncertain malignant potential (STUMP) [96]. None of these cutaneous lesions meet the regular histological criteria of any specific type of common melanocytic nevi and sporadic cutaneous malignant melanoma (SCMM). Melanocytoma usually present as solitary cutaneous lesions, though multiple melanocytoma, albeit rarely, may arise; some of these lie grouped together (agminate type) and potentially recur after removal of a solitary lesion [37,81,96]. In these cases, differential diagnosis with a primary melanoma with satellitosis is mandatory. Therefore, histological examination is mandatory, revealing cyto-architectural atypia and asymmetry, as well as an underlying inflammatory infiltration. Some melanocytoma with spitzoid features usually do not remain confined to the primary site, but also spread to other cutaneous areas, with agminated and/or satellitosis features, as reported in our case. Lymphatic involvement may be also present in melanocytoma, although we did not find any lymphatic involvement in our patient, despite the presence of multiple cutaneous satellitosis. Finally, cutaneous metastases of melanoma, as known, may present with multiple satellitoses, and they are the main differential diagnosis in case of agminated blue lesions, and/or blue lesions with satellitosis. To date, 6 different dermoscopic patterns of cutaneous melanoma metastases have been defined so far: nevus-like globular pattern, nevus-like non globular pattern, angioma-like pattern, vascular pattern and unspecific pattern [97]. However, the blue nevus-like pattern is the most common, causing important differential diagnoses with agminated (or with satellitosis) blue lesions. Usually, melanoma cutaneous metastases involve the trunk (following the primary site of melanoma), while we found that most of our agminated (and with satellitosis) blue lesions involved the head/neck region. Dermoscopically, although is impossible to differentiate cutaneous melanoma metastases from blue nevi (and other blue lesions), Bono et al. found that homogeneous, saccular and vascular patterns (showing aneurysms and winding vessels), together with pigmented halo and peripheral grey spots, seem to be the most significant elements suggestive of melanoma cutaneous metastases [98]. In any case histological analysis is needed to confirm the diagnosis, together with a clinical–pathological correlation. As reported in our Case 1, in the presence of malignant hyperpigmented lesions, an epidermal component can be present, contrariwise in benign lesions, as usually there is only a dermal proliferation.

A comprehensive list of the agminate lesions described in the literature is available below (Table 2).

## 5. Conclusions

In conclusion, we found how blue lesions may present an agminated pattern as well as a pattern characterized by multiple cutaneous satellitoses, causing important clinical, dermoscopical and pathological challenges to clinicians. As reported by our cases and by the literature, due to their depth in the dermis, currently, non-invasive imaging diagnostics for the study of blue pigmented skin lesions with agminated features and/or with satellitosis have a marginal role, while the patient’s medical history together with a histological sample remain the gold standard for the correct evaluation of these cutaneous lesions. Clinicians should be confident with these types of cutaneous lesions, to avoid misdiagnoses. This study has some limitations, such as the small sample size, but agminated blue lesions and blue lesions with satellitosis are rare in daily clinical practice; therefore, it might be hard to collect larger samples in single-center studies. Further studies, perhaps involving more centers, could be useful to expand the case studies and further increase knowledge about these rare skin lesions.

## Figures and Tables

**Table 1:** Clinical morphology, dermoscopic features and histology of the new studied lesions.

Case	Age in Years/Gender	Clinical Morphology	Dermoscopic Features	Histology
1	53/M	Blue–black plaque with multiple bluish papules and macules	Homogenous blue–gray pattern with yellow hue	Locally advanced melanoma
2	86/M	Blue–black plaque with multiple blue satellitosis	Homogenous blue–gray pattern	Metastatic melanoma
3	38/F	Blue nodule with agminated pigmentated macules	Homogenous violet-blue pattern with serpentine vessels	Desmoplastic blue nevus
4	77/F	Blue–black macule and interspersed pigmentated lesions of scalp	Homogenous blue–brown pattern	Common blue nevus

M: Male; F: Female.

**Table 2:** List of agminated blue nevi and agminated Spitz nevi described in the literature.

Author	Year	Age	Sex	Site	Cases	Dermatoscopy	Histology
Upshaw [1]	1947	9	M	Trunk	1	NP	Common blue nevus
Dorsey [2]	1954	NP	NP	NP	2	NP	Cellular blue nevus
Pittman [3]	1976	18	M	Leg	1	NP	Common blue nevus
Shenfield [4]	1980	56	M	Epigastrium	1	NP	Common blue nevus
Atherton [5]	1980	10	M	Trunk, limbs	1	NP	Common blue nevus
Hedricks [6]	1981	14	M	Sternum	1	NP	Common blue nevus
Tuthill [7]	1982	18	F	Collarbone	1	NP	Combined blue nevus
Ishibashi [8]	1990	25, 16, 15	F, F, M	Chest, leg, shoulder	3	NP	Common blue nevus
Hofmann [9]	1992	66, 36	NP	Arm, back	2	NP	Common blue nevus
Misago [10]	1993	67	M	Hypocondrium	1	NP	Combined blue nevus
Velez [11]	1993	57	M	Shoulder	1	NP	Common blue nevus
Kiene [12]	1995	29	F	Nasolabial area	1	NP	Combined blue nevus
El-Ansary [13]	2001	9	M	Sole	1	NP	Common blue nevus
Pizzichetta [14]	2007	59	F	Leg	1	Homogeneous, linear pigmented structures. Darker-sulci.	Superficial blue nevus
Chen [15]	2013	31	F	Forearm	1	NP	Common blue nevus
Milkova [16]	2013	57	F	Forehead	1	NP	Common blue nevus
Rocha [17]	2013	9	M	Thigh	1	Homogeneous pattern, diffuse brownish areas, regular network, numerous regularly distributed small dots.	Common blue nevus
Spring [18]	2013	42	F	Ear	1	NP	Fusiform and epithelioid cells
Koba [19]	2014	11	M	Sole	1	Homogeneous blue-grey pattern	Common blue nevus
Paolino [20]	2016	60	F	Forehead	1	Homogeneous blue-grey pattern	Agminated blue nevus
Paolino [20]	2016	58	M	Vertex	1	Homogeneous blue-grey pattern	Agminated blue nevus
Lisboa [21]	2016	64	M	Upper back	1	Homogeneous bluish or grayish pattern	Agminated blue naevus
Oliveira [22]	2017	15	F	Arm	1	Blue–gray in a homogenous pattern, with peripheral streaks and satellite lesions	Common blue nevus
Benson [23]	2018	19	M	Neck	1	NP	Eruptive Blue Nevus
Woo [24]	2019	49	M	Shoulder	1	Blue homogenous areas with no network pattern or structures	Agminated blue naevus and naevus spilus
Cantisani [25]	2021	31	F	Scalp	1	Homogenous blue–gray pattern, with two grey satellite lesions	Blue nevus with satellitosis
Rodríguez-Jiménez [26]	2021	52	M	Jaw	1	Homogenous blue pattern	Agminated blue nevus
Sławińska [27]	2022	52, 40, 37, 69	M, M, F, F	Scalp, Nose, Shoulder, Hand	4	Structureless blue pattern or brownish lesions	Agminated blue naevus
Hassab-El-Naby [28]	2023	NP	NP	NP	2	Central pigmented part surrounded by a well-defined hypopigmented area	Agminated halo nevus
Allen [29]	2023	67	M	Trunk	1	NP	Common blue nevus
Palazzo [30]	1988	NP	F	Scalp	1	NP	Spitz nevus
Renfro [31]	1989	5	M	Face	1	NP	Spitz nevus
Abramovits [32]	1993	12	F	Sole	1	NP	Spitz nevus
Herd [33]	1994	2, 6, 10	F, F, M	Arm, arm, arm	3	NP	Spitz nevus
Bullen [34]	1995	4,	F, F	Cheek, back	2	NP	Spitz nevus
Krasovec [35]	1995	11	M	Shoulder	1	NP	Spitz nevus
Aloi [36]	1995	40	F	Thigh	1	NP	Spitz nevus within a congenital speckled lentiginous nevus
Sabroe [37]	1996	21	F	Arm	1	NP	Spitz nevus
Hulshof [38]	1998	16	F	Shoulder, arm and hand	1	NP	Spitz nevus
Akyürek [39]	1999	19	M	Scalp	1	NP	Spitz nevus
Böer [40]	2001	16	F	Thigh	1	NP	Spitz nevus
Glasgow [41]	2005	2	M	Arm	1	NP	Spitz nevus
Aida [42]	2010	3	M	Face	1	NP	Nevus spilus with agminated Spitz nevus
Hassanein [43]	2011	5	F	Face	1	NP	Atypical spitz nevus
Zeng [44]	2012	2	M	Face	1	NP	Agminated spitz nevus
Gupta [45]	2015	60	F	Sole	1	NP	Spitz nevus
Adachi [46]	2016	23	M	Penis	1	NP	Atypical spitz nevus
Porubsky [47]	2017	14	F	Shoulder, arm	1	NP	Desmoplastic spitz nevus, nevus spilus, compound nevus
Pontoizeau [48]	2017	4	M	Arm	1	NP	Agminated spitz nevus
Van Kester [49]	2018	4	F	Foot	1	NP	Spitzoid melanoma
Adler [50]	2018	31	M	Thigh	1	NP	Spitz nevus
Nevares-Pomales [51]	2018	0	F	Face	1	NP	Agminated spitz nevus
Nemeth [52]	2018	1	F	Trunk	1	NP	Nevus spilus with agminated Spitz nevi
Robertson [53]	2021	3	F	Face	1	NP	Agminated spitz nevus
De Giorgi [54]	2021	6	M	Buttock		Irregularreticular structures, regression areas, and blue-white veil.	Spitz nevus
Wang [55]	2022	21	F	Ear	1	NP	Agminated spitz nevus
Fumero-Velázquez [56]	2023	6, 0, 42	M, F, F	Ear, foot, shin	3	NP	Atypical Spitz tumor, PRKCA fusion melanocytic tumor pigmented epithelioid melanocytoma

M: Male; F: Female; NP: Not Published.

## Data Availability

Data are contained within the article.
